# You Are Not My Handler! Impact of Changing Handlers on Dogs’ Behaviours and Detection Performance

**DOI:** 10.3390/ani8100176

**Published:** 2018-10-09

**Authors:** La Toya J. Jamieson, Greg S. Baxter, Peter J. Murray

**Affiliations:** School of Agriculture and Food Sciences, Wildlife Science Unit, The University of Queensland, Gatton Campus, Warrego Highway, Gatton 4343, Australia; gregbaxter36@gmail.com (G.S.B.); peter.murray@uq.edu.au (P.J.M.)

**Keywords:** dogs, dog handler, detection performance, behaviour

## Abstract

**Simple Summary:**

Detection dogs and their handlers must be a bonded team. Changing a dog’s handler, which occurs in certain organisations or through change of ownership, may generate team conflict and reduce detection performance. Through testing dogs at detection tasks with a familiar and unfamiliar handler, we found that dogs scored higher for detection accuracy with their familiar handler. The dogs were also less distracted with their familiar handler. These results suggest that changing a dog’s handler influences both their detection accuracy and behaviours. This may impact how working dogs are managed and their welfare.

**Abstract:**

Dog-handler relationships can directly impact team success. Changing a dog’s handler may therefore compromise detection performance. However, there are currently few studies which support this. This research explored the performance and behavioural impact of changing a dog’s handler. Nine dogs trained at scent detection were accuracy tested with a familiar and unfamiliar handler. Both handlers were female with similar dog handling experience. The dogs were tested along brick lines containing target, non-target, and control samples. Testing was separated into four sessions, with each session having 36 samples. The dogs’ accuracy scores were then calculated and testing footage behaviour coded. The dogs had significantly higher sensitivity (*p* = 0.045) and negative predictive value (NPV) (*p* = 0.041) scores when handled by the familiar handler. With the unfamiliar handler the dogs performed more stress-related behaviours, and were distracted for a higher proportion of time (*p* = 0.012). Time spent distracted was negatively correlated to detection performance (correlation = −0.923, *p* < 0.001). With the unfamiliar handler the dogs’ performance did not improve throughout testing (*p* = 0.553). This research demonstrates how these dogs’ detection performances were impacted by changing handlers. Future research is required to determine if professional dog-handler teams are impacted similarly.

## 1. Introduction

Domestic dogs (*Canis lupus familiaris*) and humans are closely bonded, with humans often replacing their dog’s conspecifics as their main social partner [1]. Due to this close relationship, dogs have been successful working partners with humans for centuries. Interactions between handlers and their animals, such as dogs and their handlers, influence their welfare and task performance [2,3,4,5,6]. There has been much research on working dogs’ abilities, but further research is needed on factors impacting their success, such as the dog’s handler and their relationship [7,8,9].

Dogs have been used by humans for detection work, which can include narcotics and wildlife detection [10,11]. Whilst a significant focus has been given to the detection dog, their handler and the dog-handler relationship has a strong influence on their working performance [6,9,12,13,14]. Handlers must be able to recognise their dog’s subtle working behaviours and assist them [15,16]. Failure to recognise these subtleties typically results in false negatives, where target samples are missed [16]. Dogs may also trust human cues over their own olfactory senses [17]. These findings demonstrate the importance of the handler on the dog’s performance and how easily they can impact their detection performance.

Dogs respond and behave differently to different people, depending on how familiar they are to each other [18,19]. For example, dogs demonstrate more ‘redirected behaviours’, including playing with inanimate objects and sniffing/licking the floor, and ‘appeasement gestures’, including blinking, averted head, and looking elsewhere, when interacting with a familiar person [18]. Dogs will also typically respond quicker to the person they have a closer relationship with [20]. Hence, it has been postulated that the ideal system for explosives detection is a single dog and single handler team [21]. However, research has typically focused on determining the importance of the dog handler’s experience, rather than the dog-handler relationship [22].

Whilst some studies have demonstrated the abilities of dogs to work with multiple handlers [23,24], no study directly compares dogs’ detection performances with an unfamiliar and familiar handler [8]. An unfortunate occurrence in the working dog community is the reluctance to share information [25]. It is therefore difficult to postulate how many working dogs experience a change of handler. Situations where this occurs include when dogs are trained and then sold to dog handlers; or when working dogs are owned by organisations or government agencies and are therefore used by multiple handlers. Belgian military dog teams, for example, are considered fully operational after a two-week settling in period with a new handler [26]. This transition period is relatively short considering it has been reported that these dogs are occasionally left in their kennels without handler interaction (excluding routine kennel cleaning and food distribution) for up to five consecutive days [27]. Changing working dog handlers may increase inconsistency or negatively affect the dog-handler bond, which is likely to generate conflict and compromise the team’s performance [1,28]. In circumstances where the dog’s role is potentially lifesaving (e.g., explosives detection), a compromised team can be catastrophic.

This preliminary study therefore aimed to compare dogs’ detection performances and behaviours when handled by a familiar and unfamiliar handler. These dogs weren’t operational working dogs, however, they had received extensive training at scent detection specifically for this research. The training principles used mimic professional detection dog training, and the dogs’ detection accuracy was rigorously assessed. We hypothesised that: (1) the dogs would have higher mean accuracy scores with the familiar handler than the unfamiliar handler; and (2) the dogs would perform more stress-related behaviours, and be more distracted, when handled by the unfamiliar handler.

## 2. Materials and Methods

### 2.1. Research Dogs

Nine dogs extensively trained in scent detection work were used in this project (Table 1). These dogs were sourced from dog breeders, private owners, and an adoption centre. This research is part of a larger breed comparison training project where three dog breeds were used—Border Collies, Labrador Retrievers, and Greyhounds. After a literature review [29], Border Collies were selected as they are perceived to have the most suitable behavioural and physical traits for detection work, whilst Greyhounds were perceived to have the least. Labrador Retrievers were selected as they are one of the most commonly used breeds for detection work. These dogs were not professional detection dogs; however, they had received three months detection training, five days per week, before testing. This training used operant conditioning, with a significant focus on positive reinforcement, e.g., Reference [30], to make the dogs associate their target scent with their reward (food). This training mimicked how professional working dogs are trained and is therefore comparable. Once the dogs were consistently making this association, non-target samples were included in the samples presented to improve their odour discrimination ability. The dogs’ training used a similar sample layout as described in Section 2.3. These dogs were only tested if they achieved a high level of detection proficiency in training. Three dogs were therefore not included in this study as they did not reach this level of detection proficiency. Prior to testing, the dogs’ behaviours were assessed using the Match-Up II Shelter Dog Rehoming Program/Behavior Evaluation [31]. From this assessment the dogs’ behaviour scores for ‘Friendliness’, ‘Fearfulness’, ‘Excitability’, ‘Aggressiveness’, ‘Playfulness’, and ‘Trainability’ were calculated. These scores were based on the dogs’ performance and frequency of related behaviours (e.g., sniffing, licking, or nudging a person were related to their ‘Friendliness’ score). Three behaviour assessment sub-tests were not completed as they weren’t relevant to this study. Adjustments were therefore made to the behaviour assessment totals. This research had the University of Queensland’s Animal Ethics Committees approval (approval number: SAFS/454/16) to house, train, and accuracy test all involved dogs.

### 2.2. Dog Handlers

Both dog handlers were female, of similar height and build, with similar dog handling experience. Neither handlers were professional dog trainers/handlers, however, both had previously been instructed in dog training, either their own or other dogs, for several years. Handler 1 was the dogs’ trainer throughout this project. Handler 2 was introduced to the dogs on the first day of testing. Prior to testing, Handler 2 was sent information on each dog. This included basic information about the dogs, their personality, and tips on handling them (Appendix A). The morning of the first testing session, Handler 2 was also instructed by Handler 1 on how to handle each dog. Handler 2 had several practise runs along a mock test line-up with the dogs, whilst being instructed by Handler 1, for approximately 30 min. It was presumed in a real-world setting that a professional detection dog would not be transferred to another handler without this information being provided and this guidance given.

### 2.3. Testing Layout

Accuracy tests were completed to measure each dog’s detection performance with both handlers. These tests were completed outdoors in a paddock used for grazing cattle, but none were present during testing. Tests were completed outdoors to better mimic the detection requirements of operational detection dogs. Clay house bricks (33 × 8 × 12 cm) with eight holes in each were laid out at a measured distance along a straight line. Each line comprised 18 bricks, which were separated into three groups of six. Each brick in a group was 2 m apart and there was 5 m between each group of six bricks. Target, non-target, and control samples were presented in the holes of these bricks. The target sample was Bengal tiger (*Panthera tigris tigris*) scat, and the non-targets were cow (*Bos taurus*) and Brush-tailed phascogale (*Phascogale tapoatafa*) scat. These samples were collected from captive facilities and a rural property, and were stored at −20 °C in a freezer. All samples were contained in 5 mL plastic vials which, for storage and transport, had a screw cap. Control bricks and control samples were used in all brick lines, with the control samples being a brick with an open, but empty, vial. Of the 18 bricks, two were targets, nine were non-targets, four were controls, and three were empty bricks. The samples were randomly allocated to bricks, with eight brick lines being constructed over two testing days. The handlers did not know the order of the samples during testing. All dogs were tested along the same two brick lines in each session, with their testing order randomised.

Care was taken to minimise the likelihood of cross-contamination between the samples. Each species’ samples were only placed in their own specific bricks. Prior to testing these bricks were sterilised in boiling water, sun dried, and placed into new 50 L plastic storage tubs for storage and transportation. Labelled gloves, specific to each species, were worn when handling the samples or their bricks. The target and non-target samples were stored, transported, and handled separately, by separate field assistants.

### 2.4. Testing Procedures

Prior to the dogs’ training, their testing order was randomly drawn. Five dogs (Dogs 1, 3, 6, 7, and 9) were tested firstly with Handler 1, whilst four dogs (Dogs 2, 4, 5, and 8) were tested firstly with Handler 2. After the first tests were complete (four sessions with each handler), the dogs had a rest day before being tested with the other handler. All tests therefore took four days to complete, with two testing sessions per day (morning and afternoon). Wind conditions, air temperature, and humidity levels were collected from the Australian Government’s Bureau of Meteorology weather station near to the testing site (Appendix B).

During testing, the other handler and dogs were a minimum of 50 m away from the team being tested, down wind and out of sight. The dog and handler team would walk along the brick line, with the handler repeating the cue ‘find’. A recording observer, who followed the dog team at approximately 5 m distance, was responsible for recording their indications and informing the handler if the indication was correct. The handlers were unknowing of the sample order. A person followed the recording observer at a 10 m distance from the dog-handler team and filmed all testing. The dogs indicated by sitting and facing the handler, or by stopping and turning to face the handler at the target brick. The dogs were rewarded with food and verbal praise. The dogs were not rewarded if they falsely indicated. To account for the changing and unpredictable wind conditions during testing, the dogs were allowed up to three attempts at each brick line. If the dogs did not locate the target by the third attempt the test ceased. By testing conclusion, each dog and handler team had been tested with 144 samples during four test sessions.

### 2.5. Behaviour Coding

All tests were recorded using a GoPro Hero4 silver camera. The dogs’ behaviours were analysed and coded using Behavioral Observation Research Interactive Software (BORIS, [32]). Continuous behavior sampling was completed. Stress-related behaviours were the main behaviours coded, which measured both their occurrence, frequency, and duration. Behaviours coded were lip licking, tail lowering, ears pinned back, yawning, whining, jumping, and shaking off [33]. Behaviours were removed from future analyses if less than 50% of the dogs performed the behaviours at least once (e.g., whining). The proportion of time the dogs spent ‘distracted’ or ‘scenting’ was also calculated. ‘Distracted’ was defined as when the dog was ignoring the handler’s commands in order to smell or view other stimuli. ‘Scenting’ was defined as when the dogs were actively scenting/smelling along the brick line. At the conclusion of behaviour coding, time budgets were created.

### 2.6. Data Analyses

From the dogs’ positive, negative, and false indications, their sensitivity, specificity, positive predictive values (PPV), and negative predictive values (NPV) were calculated. Sensitivity is a dog’s ability to locate their target and specificity is their ability to identify and not indicate to a non-target [34]. These are commonly used measurements of a detection dog’s accuracy [35]. A dog’s PPV assesses the correct proportion of their target-present indications and NPV assesses the correct proportion of their target-absent indications [34]. General linear models were constructed to determine any relationships between the environmental conditions (wind speed, air temperature, and humidity) and the dogs’ performance. Statistical significance was set at <0.05. Spearman correlations were completed to determine the relationship between the dogs’ ages and their accuracy scores. Spearman correlation was used as the data was not linear. Two-sample *t*-tests were completed to determine if there was a significant difference between the dogs’ performances with Handler 1 and Handler 2. General linear models were constructed to determine the impact of the training group and the testing session on the dogs’ performances. Two-sample *t*-tests were completed to determine the difference between the dogs’ behaviours when handled by the different handlers. Pearson correlations were completed to determine the relationship between the proportions of time spent ‘distracted’ and ‘scenting’ and the dogs’ performances.

## 3. Results

The dogs (*n* = 9) had significantly higher sensitivity (*p* = 0.045) and NPV scores (*p* = 0.041) with Handler 1 (Table 2). When the dogs were with Handler 1 they also had higher mean PPV scores than when they were with Handler 2 (80.9 and 53.5, respectively), however this was not statistically significant (*p* = 0.114). There was no significant difference between the dogs’ specificity scores between the handlers (*n* = 2). Of the nine dogs, three did not work for Handler 2, as demonstrated by their sensitivity and PPV scores of zero (Table 2).

**Figure 1:**
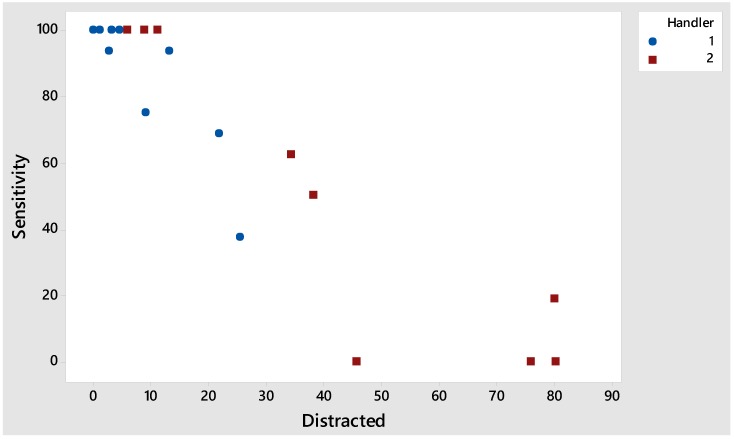
Strongly correlated negative relationship between the proportion of time (%) spent ‘distracted’ and the dogs’ sensitivity scores with Handler 1 and 2. The dogs’ breeds are also included.

The dogs also behaved differently with the two handlers. The dogs had their ‘tails lowered’ (*p* = 0.035) and were ‘distracted’ (*p* = 0.012) significantly more when handled by Handler 2. The proportion of time the dogs spent ‘distracted’ was significantly higher with Handler 2 (*p* = 0.012), which significantly affected their sensitivity (*p* = 0.004), PPV (*p* = 0.010), and NPV scores (*p* = 0.005). The proportion of time spent ‘distracted’ had a strong relationship with the dogs’ sensitivity (correlation = −0.923, *p* < 0.001; Figure 1), PPV (correlation = −0.846, *p* < 0.001), and NPV (correlation = −0.925, *p* < 0.001) scores.

**Figure 2:**
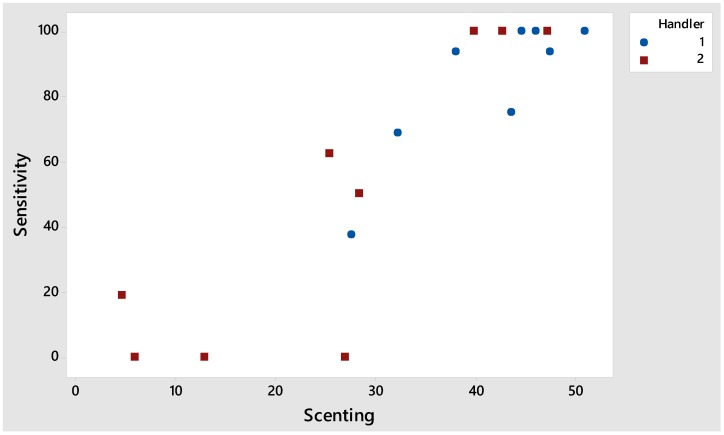
Strongly correlated relationship between the proportion of time (%) spent ‘scenting’ and the dogs’ sensitivity scores with Handler 1 and 2.

The dogs spent a significantly higher proportion of their time ‘scenting’ (*p* = 0.022) with Handler 1, which significantly influenced the dogs’ sensitivity (*p* < 0.001; Figure 2), PPV (*p* = 0.003), and NPV (*p* < 0.001) scores. Proportion of time spent ‘scenting’ was strongly correlated to the dogs’ sensitivity (Pearson correlation = 0.897; *p* < 0.001).

The dogs’ sensitivity significantly improved during the testing sessions when handled by Handler 1 (*p* = 0.017). The dogs’ sensitivity did not improve through the testing sessions with Handler 2 (*p* = 0.553), and there was a very weak relationship between testing session and sensitivity (Pearson correlation = 0.029; *p* = 0.867). There was a weak relationship between the dogs’ age and their sensitivity (Spearman correlation = 0.099, *p* = 0.697), specificity (Spearman correlation = −0.383, *p* = 0.117), PPV (Spearman correlation = −0.209, *p* = 0.406), and NPV scores (Spearman correlation = −0.056, *p* = 0.825). There was no significant difference between the three testing groups, based on the dogs’ sensitivity (*p* = 0.088), specificity (*p* = 0.409), PPV (*p* = 0.157), and NPV (*p* = 0.080) scores. The dogs’ detection performance was also not significantly impacted by the environmental conditions (wind speed, *p* = 0.185; air temperature, *p* = 0.835; or humidity, *p* = 0.641).

There were evident differences between the dogs’ behaviour assessment scores (Table 3). There were also few apparent similarities between the dogs who worked well for both handlers.

## 4. Discussion

The variation between the dogs’ detection performances with Handler 1 and Handler 2 was significant. The dogs had significantly lower accuracy scores when handled by Handler 2. Whilst the dogs continued to improve throughout their testing with Handler 1, there was no improvement with Handler 2. Due to the physical and dog handling experience similarities between the handlers, it can be assumed that this variation was at least in part influenced by Handler 1′s familiarity with the dogs. Familiarity may however, not be the only influential factor. As a result of training the dogs, it can be presumed that Handler 1 was also strongly bonded with the dogs, and they with her. This bond and frequent contact have previously been correlated with higher team performance [9,36]. As proposed by Horn et al. [20], familiarity may not be as influential as a strong dog-handler relationship on the dog’s performance or learning ability. This may also be the reason for the dogs’ lack of improvement with Handler 2. The fact that three of the nine dogs also refused to work for Handler 2 further supports this. The lack of improvement with Handler 2 is concerning for professional working dogs that are transferred between handlers. This is especially true when the transition period has time restrictions (e.g., two weeks until a detection team is fully operational [26]). It is likely that within a certain time the dogs would become familiar and eventually bond with Handler 2. However, there is no knowing the time this would take or the level of impact this would have on their performance. This acclimatisation time would also likely vary depending on the personalities of both the dogs and handlers. This requires further research.

There were little similarities between the dogs who performed well with both handlers. Of these three dogs, one was a male Border Collie and two were female Labrador Retrievers. We are not suggesting this was breed related, although further research is warranted. There were no consistent similarities in their behaviour scores. The only similarity between these dogs is their high scores with Handler 1. This indicates that perhaps dogs who are high performers will transfer more easily to a new handler. This was not consistent with all the dogs however, as there were other high performing dogs (Dog 2 and 4) who performed very poorly for Handler 2. A larger sample size may be needed to highlight the characteristics of dogs that can adjust more quickly to new handlers, and this should be researched further.

Along with their differences in performance, the dogs also behaved differently with the two handlers. With Handler 2 the dogs demonstrated more stress-related behaviours and were significantly more ‘distracted’. The dogs’ lack of focus with an unfamiliar handler has been replicated in other studies [20,37]. The dogs also spent less time ‘scenting’ with Handler 2, which was influential on the dogs’ performances. The dogs’ specificity scores were likely not impacted by their time spent ‘distracted’ or ‘scenting’ as they could achieve a score of 100 even if they weren’t scenting, simply by never indicating. This is demonstrated by Dogs 4, 5, and 8 all having sensitivity scores of 0 (meaning they never indicated to a target), but having specificity scores of 98.4, 100, and 99.2, respectively. This highlights the importance of not evaluating dogs’ detection performances based solely on one calculation.

This study was limited by a small sample size. Due to the extended time commitment and resources needed to kennel and train the dogs, this could not be avoided. Even with this small sample size, the impact of the dog handler and the dog-handler relationship was clearly evident. This impact may not be as significant if professional working dogs and handlers were used, and this should be explored further. It is likely, however, that even professional working dogs are impacted by a change of handler, whether through their behaviours, and therefore their welfare, or working performance.

## 5. Conclusions

This study has demonstrated the behavioural and performance impact of changing a dog’s handler. Our results may therefore not only have implications for detection dogs, but all dogs required to work closely with humans (e.g., assistance and herding dogs). Whilst each dog was impacted differently by this change, collectively the dogs responded negatively to the change of handler. It is unclear how long it would take for the dogs to adjust to a new handler and the best ways to manage this transition should be researched further. Whilst this research had a small sample size and only demonstrates how these specific dogs were affected by changing these specific handlers, our results and their possible outcomes are still significant and are further supported by the literature. This research highlights that whilst dogs are an incredible working partner for humans, they are not simple minded, easily transferable machines, and should not be managed as such.

## Figures and Tables

**Table 1:** Detection dogs used during this project. Due to their extended training time, these nine dogs were separated into three groups: March–June (Group 1), June–September (Group 2), and September–November 2017 (Group 3).

Group	Dog	Sex	Neuter Status	Age (Years)	Breed
1	1	Male	De-sexed	6	Border Collie
1	2	Female	Entire	2	Border Collie
1	3	Female	Entire	2.5	Labrador Retriever
2	4	Female	Entire	4	Border Collie
2	5	Male	Entire	2	Labrador Retriever
2	6	Female	Entire	2	Labrador Retriever
3	7	Male	Entire	4	Border Collie
3	8	Female	Entire	5	Labrador Retriever
3	9	Female	Entire	3.5	Greyhound

**Table 2:** Sensitivity, specificity, PPV and NPV scores with Handler 1 and Handler 2. Mean scores and standard deviations (SD) are also provided. Dogs 4, 5, and 8 did not work for Handler 2 as demonstrated by their scores of zero for sensitivity and PPV.

Dog	Handler	Sensitivity	Specificity	PPV	NPV
1	1	100	95.3	72.7	100
	2	100	99.2	94.1	100
2	1	100	100	100	100
	2	50	97.7	72.7	94
3	1	100	95.3	72.7	100
	2	100	100	100	99.2
4	1	93.8	100	100	99.2
	2	0	98.4	0	88.7
5	1	37.5	97.7	66.7	92.6
	2	0	100	0	88.9
6	1	100	100	100	100
	2	100	99.2	94.1	100
7	1	68.8	92.2	52.4	96
	2	18.8	96.1	37.5	90.4
8	1	75	96.8	75	96.8
	2	0	99.2	0	88.8
9	1	93.8	98.4	88.2	99.2
	2	62.5	98.4	83.3	95.5
Mean	1	85.4	97.3	80.8	98.2
	2	47.9	98.6	53.5	93.9
SD	1	21.4	2.6	17.1	2.5
	2	44.8	1.2	44.1	4.9

**Table 3:** The dogs’ behaviour assessment scores for Friendliness (score/23), Excitability (score/23), Playfulness (score/17), Fearfulness (score/24), Aggressiveness (score/24), and Trainability (score/15). The dogs who performed well for both handlers are in bold.

Dog	Friendliness	Excitability	Playfulness	Fearfulness	Aggressiveness	Trainability
**1**	**14**	**6**	**0**	**6**	**2**	**12**
2	9	1	1	2	0	15
**3**	**23**	**14**	**8**	**2**	**0**	**15**
4	5	1	1	3	0	9
5	20	7	7	5	0	9
**6**	**17**	**2**	**1**	**6**	**0**	**8**
7	20	9	5	1	0	12
8	18	6	7	3	0	8
9	19	1	8	2	0	5
**Mean**	**16.1**	**5.2**	**4.2**	**3.3**	**0.2**	**10.3**

## Appendix A

Below is an example of the information provided to Handler 2 about one of the dogs she handled. This level of detail was provided for the description of all nine dogs. This information was provided a minimum of one week prior to training and in this time Handler 2 was able to ask any necessary questions.

**General information**

Jasper is a 6-year-old Border Collie who has learnt detection work incredibly quickly. He is very eager to learn and please, however, he is quite timid around people. I have introduced him to most of the project volunteers during training and whilst he isn’t overly happy around them, he typically isn’t distracted by them when working. When he is working he is a very different dog—very energetic and enthusiastic. Jasper does have a beautiful nature, but please remember he is sensitive.

**Handling tips**

Jasper is simple to handle and once he is on task he is very focused. He rarely falsely indicates and has almost never missed a target in training. His only quirk is that on occasion he gets too excited to work—he jumps up-and-down in front of you when walking along the bricks (containing the samples). Simply say in a low tone, “Jasper, find” and do a sweeping motion with your hand along the brick line. I typically try to use a soothing, encouraging tone of voice with him, and do not raise my voice around him. Even though it is tempting, Jasper does not really enjoy being patted and it sometimes causes him stress if strangers try to pat him (his ears go back, he freezes or turns his head and lip licks). As a result, if you notice Jasper is becoming anxious try talking to him softly and possibly distance yourself from him. He may alternatively move and sit behind you if scared or intimidated by something/someone. If you are concerned he is becoming too overwhelmed being handled by yourself please let me know. I do not believe, however, that this is likely.

## Appendix B

**Table A1:** Mean environmental conditions experienced on testing days.

Group	Day	Session	Windspeed (km/h)	Air Temperature (°C)	Air Humidity (%)
1	1	1	15	14.1	52
		2	14.5	18.6	63
	2	1	9.5	22.9	33
		2	13	19.2	41
	3	1	10	12.5	70
		2	14.5	17.2	58
	4	1	9.5	22	40
		2	7.5	17.3	52
2	1	1	11	22.9	52
		2	28	31	21
	2	1	19	20.1	22
		2	26	26.2	15
	3	1	7	16.2	57
		2	11	24.4	30
	4	1	6	18	54
		2	13	27.4	18
3	1	1	12	25	60
		2	21.5	29.2	45
	2	1	14	25.7	55
		2	14	30.4	42
	3	1	15	26.2	60
		2	17	30.3	44
	4	1	17	24.6	73
		2	22	23.2	81
